# Comparing the Effects of Concord Grape (*Vitis labrusca* L.) Puree, Juice, and Pomace on Intestinal Morphology, Functionality, and Bacterial Populations In Vivo (*Gallus gallus*)

**DOI:** 10.3390/nu14173539

**Published:** 2022-08-27

**Authors:** Nikita Agarwal, Viral Shukla, Nikolai Kolba, Cydney Jackson, Jacquelyn Cheng, Olga I. Padilla-Zakour, Elad Tako

**Affiliations:** Department of Food Science, Cornell University, Stocking Hall, Ithaca, NY 14850, USA

**Keywords:** grape pomace, stilbenes, intra-amniotic administration, poultry feed, gut microbiome, brush border membrane, in-ovo, nutrition

## Abstract

This is a preliminary study evaluating the effect of different fractions of Concord grapes (*Vitis labrusca* L.) on the brush border membrane (BBM) morphology, duodenal gene expression, and specific gut bacterial populations. For this study, we utilized a unique intraamniotic approach, wherein, the test substances are administered into the amnion of the *Gallus gallus* egg (on day 17). The embryo orally consumes the amniotic fluid along with the injected test substance before the hatch. We randomly divided ~50 fertilized eggs into 5 groups including 6% grape (juice, puree, and pomace) along with controls (no injection and diluent—H_2_O). The grape juice was prepared by crushing the grapes; the grape residues were used as pomace. The grape puree included the grape skin, endocarp, mesocarp, and juice but not the seeds. On day 21, the hatch day, the blood, pectoral muscle, liver, duodenum, and large intestine were harvested. Our results showed no significant differences in blood glucose, pectoral glycogen level, or body weight. However, significant (*p* < 0.05) differences in duodenal and liver gene expression were observed between the treatment groups. The grape puree treatment resulted in higher *Clostridium* numbers and lower *Bifidobacterium* numbers when compared to all other groups. In summary, the dietary consumption of grape polyphenols has the potential to beneficially modulate aspects of intestinal health provided their concentration is limited.

## 1. Introduction

The Concord grape is native to North America and was first propagated in New York State in the 1870s [1]. The state currently is the second-largest producer (after Washington) and houses the largest Concord grape industry in the country [2]. The demand for grapes and their products was 53.8 million in 2020 in the United States alone [3]. The grape industry squeezes out the juice from the fruit leaving behind the skin, seeds, and flesh. These by-products, collectively referred to as grape pomace (GP), account for 25% of the harvested grape by weight [4]. Grape pomace has a high concentration of health-promoting polyphenols with a low pH making GP resistant to biological degradation [5]. Currently, the use of GP is inadequate, with most of it being dumped in landfills causing economic and environmental problems [6]. Grape puree (GPR), consisting of skin, flesh, and juice, is a by-product of grape seed extracts [7]. The objective of this study was to compare the different grape fractions—puree, juice, and pomace for their effects on intestinal morphology (villi surface area, goblet and Paneth cell number, and diameter), functionality (duodenal gene expression), and gut microbial populations, in-vivo (*Gallus gallus*).

The bioactives and micronutrients are concentrated in different fractions/matrices of the grape [8]. Another red grape variety (Bordo) was found to have the majority of its hydroxybenzoic acids (including gallic, syringic, and ellagic), hydroxycinnamic acids, flavonol- epicatechin, proanthocyanidin—B2, flavonols (myricetin, quercetin), and anthocyanins (cyanidin, delphinidin, malvidin, and peonidin) concentrated in the peel compared to its pulp or seed [8]. As the pulp fraction is constituted of ~80% water, most of the micronutrients and crude fiber are found in seed and peel [9]. Grape seeds are said to have 11% fiber, 3% minerals, 35% fiber, and only 7% water [10]. Given the nutritional composition, low cost, and sustainability aspects of utilizing GP, numerous studies have been conducted to valorize GP [5,6,11].

Pharmacologically, grape skin has been said to have antioxidant [12,13], cardio-protectant [14], anticancer [15,16], anti-inflammatory [17,18], and anti-microbial activity [19,20]. Whereas grape seeds and grape seed extracts have been extensively studied and said to be anti-diabetic, antioxidant, anti-platelet, anti-cholesterol, anti-inflammatory, anti-aging, anti-neurodegenerative, and anti-microbial agents [21,22,23,24]. In the present study, however, we were focused on comparing grape fractions for their effects on molecular, morphological, and microbial aspects of intestinal health, in-vivo (*Gallus gallus*).

Diet is one of the major factors that influence gut microbiota [25,26]. The microbiota in turn affects digestion and nutrient absorption, among others [27]. The microbes ferment the food and produce a range of metabolites as by-products including amino acids, short-chain fatty acids, and enzymes, among others [28]. These metabolites are in direct contact with the cells of the brush border membrane (BBM) including goblet cells, Paneth cells, and enterocytes affecting their proliferation [29]. The increased proliferation of enterocytes leads to an increase in absorptive (villus) surface area which in turn aids in efficient digestion and nutrient absorption, among others. On the other hand, goblet and Paneth cells are secretory cells producing mucin and anti-microbial peptides, respectively. Mucin forms the mucus layer that houses the microbiome, and the peptides regulate the relative abundance of the resident bacterial populations [30].

The *Gallus gallus* in-vivo model is a physiologically relevant model for assessing the effects of bioactive compounds on humans as the two have similar intestinal morphology, microbiota (phylum-level), and certain metabolism-related genes [31,32,33,34,35,36,37,38,39,40]. Here we utilize the embryonic phase of the bird, closed off from the external environment (in the egg) with the only variable being the injected treatment. Followed by multi-level analysis to potentially uncover the mechanism by which these grape fractions may benefit health.

## 2. Materials and Methods

### 2.1. Sample Preparation

**Figure 1:**
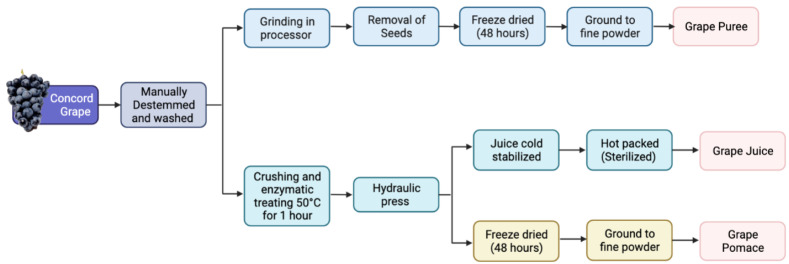
Depicts the processes carried out to prepare the grape puree, pomace, and juice. The Concord grapes were hand-picked, manually destemmed, and washed. The juice was cold pressed followed by sterilization. The grape puree and pomace were freeze-dried. The resultant fractions included: puree (skin + juice + flesh), juice, and pomace (skin + seeds + flesh).

Concord grapes (*Vitis labrusca* L.) were picked locally and processed at the Cornell Food Venture Center Pilot Plant (Geneva, NY, USA). Grapes were manually destemmed and washed. The grape puree (GPR) was made by grinding whole grapes in a bench-scale processor (Robo Coupe; Jackson, Mississippi) for 30 s at 1500 RPM. Seeds were manually removed, and the resulting pulp was then freeze-dried (Max53, Millrock Technology, Kingston, NY, USA) for 48 h. The dried mass was then ground into a fine powder using a bench-scale processor. Grape juice (GJ) was made to mimic typical industry standards. GJ was made by pre-crushing and enzymatically treating (Rapidase^®^ added at 0.2 mL/kg, DSM Food Specialties USA, Inc., South Bend, IN, USA) the grapes at 50 °C for 1 h. They were then pressed using a pilot-scale hydraulic press (Orchard Equipment Co., Conway, MA, USA) at 8.27 × 10^6^ —9.64 × 10^6^ Pa. The resulting juice was cold stabilized at 2 °C for 48 h. The juice was commercially sterilized by hot-packing into bottles at 85 °C for 2 min (Microthermics; Raleigh, North Carolina, USA). Grape pomace (GP) was made by taking the resulting pomace from the juice pressing and freeze-drying for 48 h. The dried mass was ground into a fine powder using a bench-scale grinder. The powders were vacuumed sealed, and all samples were kept frozen until use. Figure 1 depicts the processes carried out.

### 2.2. Polyphenols and Carbohydrate Analysis

#### 2.2.1. Grape Sample Preparation

Grape samples were extracted using absolute methanol under dark and constant agitation for 2 h. The resulting slurry was centrifuged and decanted to obtain the supernatant. The resulting extract and washings were diluted in distilled water to achieve a 15% *w*/*v* extract which was used for further analysis as below.

#### 2.2.2. Polyphenol Analysis

Total polyphenol content (TPC) was determined using the Folin-Ciocalteu method as described by Waterhouse [41]. Briefly, the extract was reacted with Folin-Ciocalteu reagent and allowed to incubate at room temperature. The reaction was then quenched with sodium carbonate solution. The samples were then immediately measured for absorbance at 765 nm using a UV-visible spectrophotometer (Thermo Fisher; Waltham, MA, USA). TPC was calculated as gallic equivalents (GE) using a standard curve prepared under the same conditions.

Total monomeric anthocyanin (MA) content was determined using the pH differential method [42]. Briefly, extracts were diluted with pH 1.0 (0.025 M potassium chloride) and pH 4.5 (0.4 M sodium acetate) buffers and allowed to incubate at room temperature for 20 min. Absorbance was measured at 520 nm and 700 nm using a UV-visible spectrophotometer. Total MA content was calculated as cyanidin-3-glucoside equivalents (CE) using the equation below:

((A520, pH1 − A700, pH1) − (A520, pH4.5 − A700, pH4.5)) × 529 × dilute factor × 1000/28,000

#### 2.2.3. Fibrous and Non-Fibrous Carbohydrate Analysis

The non-fibrous carbohydrate analysis (NFC) was conducted according to AOAC 962.09. Acid detergent fiber (ADF) and neutral detergent fiber (NDF) analyses were conducted according to 973.18. The analysis was performed by Dairy One Co-Op Inc (Ithaca, NY, USA). 

### 2.3. Animals and Study Design 

Fertile Cornish Cross broiler eggs (*n* = 60) were purchased from a commercial hatchery (Moyer’s Chicks, Quakertown, PA, USA). The eggs were incubated under optimal conditions at the Cornell University Animals Science poultry farm incubator. All animal protocols were approved by the Cornell University Institutional Animal Care and Use Committee (ethic approval code: 2020-0077). 

#### 2.3.1. Water Extract Preparation

The grape pomace, puree, and juice were further extracted and diluted (in water) before they were administered into the amniotic fluid of the egg. The osmolarity of 4,6,8, and 10% solutions were tested to ensure the value was below 320 Osm/kg H_2_O and 6% was selected. The day before injection (i.e., day 16 of embryonic incubation) the respective 6% solutions were prepared in distilled water. The solutions were immersed in a water bath at 60 °C for 60 min. The solutions were centrifuged, and the supernatant was collected and stored at −20 °C until the next day.

#### 2.3.2. Intra-Amniotic Administration and Sample Collection

On day 17 of embryonic incubation, the prepared water extracts (Section 2.3.1) were thawed at 21 °C for 1 h and then placed alongside the eggs in the incubator. Candling was used to distinguish the viable eggs, and the non-viable eggs were appropriately discarded. The viable eggs were weighed and randomly distributed into 5 groups with approximately 10 eggs each. The amniotic fluid, i.e., the injection spot, was determined using candling and was marked. A 1 mL aliquot of the water extracts was then injected, respectively, using a 21-gauge needle and covered with cellophane tape. The 5 groups were assigned as follows: controls, (1) no injection, (2) H_2_O and treatments all 6% solutions of (3) GJ (4) GP, (5) GPR. The injected eggs were placed in hatching baskets in the incubator until hatch day, i.e., day 21. Soon after hatching, the chicks were euthanized in a CO_2_ chamber. The blood, pectoral muscle, duodenum, cecum, and liver were collected. The blood and pectoral muscle were placed on ice, whereas the other tissues were immediately kept in liquid nitrogen. The samples were shifted to −20 °C until further analysis.

### 2.4. Blood Glucose Measurements 

Blood was collected in 1.5 mL Eppendorf tubes and stored at 4 °C until analysis. The Accu-Chek^®^ blood glucose monitor (Roche, Indianapolis, IN, USA) was used to determine blood glucose levels. Exactly 0.6 µL of blood was placed on the disposable electrochemical test stripe. The digitally displayed reading corresponding to each sample was recorded.

### 2.5. Pectoral Muscle–Glycogen Content

Pectoral muscle samples were weighed (20 ± 1 mg) and homogenized in 8% perchloric acid following the method described by Dreiling et al. [43]. Glycogen was estimated using spectroscopy measuring the wavelength at 450 nm and calculated against a standard curve [36].

### 2.6. Gene Expression Analysis

#### 2.6.1. Isolation of Total RNA from Duodenum and Liver Tissue Samples

Total RNA was isolated using Qiagen RNeasy Mini Kit (RNeasy Mini Kit, Qiagen Inc., Valencia, CA, USA) following the manufacturer’s protocol. Briefly, 30 ± 2 mg of the proximal duodenal tissue and liver tissue (*n* = 5) were weighed in 2 mL tubes. RLT^®^ (plus β-mercaptoethanol) was added to each tube and a rotor-stator homogenizer (Omni International, Inc, Kennesaw, GA, USA) was used to disrupt the tissue samples. The lysate so obtained was centrifuged at 8000× *g* for 3 min at 20 °C. The supernatant was transferred to a new tube and 700 µL of 70% ethanol was added to each tube. The sample was then run through the RNeasy kit’s mini-column and centrifuged at 8000× *g* for 15 s. The flow-through was discarded. To this, 500 µL of RPE^®^ buffer was added and centrifuged at 8000 × *g* for 15 s. This step with RPE^®^ was repeated for 2 min. The filter part of the column alone was transferred to a new 1.5 mL collection tube and 50 µL of RNase-free water was used to elute RNA from the filter into the tube. Eluted RNA was stored at −80 °C until analysis. All steps were carried out in an RNase-free environment. RNA was quantified using a spectrophotometer at 260/280 nm. Gel electrophoresis (EtBr stain) was used to verify the integrity of the RNA obtained. DNA contamination was removed using TURBO DNase removal kit (AMBION, Austin, TX, USA).

#### 2.6.2. Real-Time Polymerase Chain Reaction (RT-PCR)

cDNA was created using the 20 µL reverse transcriptase (RT) reaction, performed in a BioRad C1000 Touch Thermocycler using the Improm-II Reverse Transcriptase Kit (Promega, Madison, WI, USA). The concentration of cDNA was assessed using Nanodrop (Thermo Fisher Scientific, Waltham, MA, USA). Further details can be found in a previous publication [31].

#### 2.6.3. Primer Design

The primers were designed based on gene sequences from the GenBank database, and the Real-Time Primer Design Tool software (IDT DNA, Coralville, IA, USA) was used [32]. The primer sequences related to iron, zinc, Vitamin A metabolism, immune response, and brush border membrane functionality that was used in this study are summarized in Table 1. The specificity of the primers was verified using the BLAST search against the genomic National Center for Biotechnology Information (NCBI) database. The reference gene used was the 18S rRNA specific for the *Gallus gallus* model.

#### 2.6.4. Real-Time qPCR Design 

All procedures were conducted as previously described [36,38,39,44]. Briefly, cDNA was used for each 10 µL reaction together with 2×BioRad SSO Advanced Universal SYBR Green Supermix (Hercules, CA, USA) which included buffer, Taq DNA polymerase, dNTPs, and SYBR green dye. Specific primers (forward and reverse) (Table 1) and cDNA or water (for no template control) were added to each PCR reaction. For each gene, the optimal MgCl_2_ concentration produced the amplification plot with the lowest cycle product (Cp), the highest fluorescence intensity, and the steepest amplification slope. Master mix (8 µL) was pipetted into the 96-well plate and 2 µL cDNA was added as a PCR template. Each run contained 7 standard curve points in duplicate. A no-template control of nuclease-free water was included to exclude DNA contamination in the PCR mix. The double-stranded DNA was amplified in the Bio-Rad CFX96 Touch (Hercules, CA, USA) using the following PCR conditions: initial denaturing at 95 °C for 30 s, 40 cycles of denaturing at 95 °C for 15 s, various annealing temperatures according to Integrated DNA Technologies (IDT) for 30 s and elongating at 60 °C for 30 s. 

The data on the expression levels of the genes were obtained as Cp values based on the “second derivative maximum” (automated method) as computed by Bio-Rad CFX Maestro 1.1 (Hercules, CA, USA). For each evaluated gene, the reactions were run in duplicate. All assays were quantified by including a standard curve in the real-time qPCR analysis. The next four points of the standard curve were prepared by a 1:10 dilution, in duplicate. A graph of Cp vs. log 10 concentrations was produced by the software and the efficiencies were calculated as 10[1/slope]. The specificity of the amplified real-time RT-PCR products was verified by melting curve analysis (60–95 °C) after 40 cycles, which should result in several different specific products, each with a specific melting temperature. 

### 2.7. Intestinal Bacterial Population Assessment

#### 2.7.1. Intestinal Sample Collection and DNA Extraction

The cecum samples (*n* = 5) were weighed (0.2 ± 0.02 g) under aseptic conditions. Details of the experiment can be found as previously described [31]. The tissues were sheared using beads and a vortex. EDTA and lysozyme were used for DNA extraction in addition to the Wizard Genomic DNA purification kit (Promega Corp., Madison, WI, USA).

#### 2.7.2. Primer Design and PCR Amplification

Primers for genus *Clostridium*, *Bifidobacterium*, *Lactobacillus*, *Klebsiella,* and species *E. coli* and *L. plantarum* were designed as per previous literature [45,46,47,48]. The primers used in this study are detailed previously [49]. All known bacteria were identified using a Universal primer. The PCR amplification was carried out by adding the DNA extracted (as template) to a PCR premixture containing nuclease-free water, PCR buffer, Taq polymerase, dNTPs, and primer. PCR conditions were set as previously optimized [49]. The PCR products were quantified using gel electrophoresis with ethidium bromide. Visualized in Gel-Pro analyzer version 3.0 (Media Cybernetics LP, Rockville, MD, USA) [47].

### 2.8. Morphometric Examination of Duodenal Tissue

The duodenal morphological examination was conducted as previously described [31]. Briefly, the duodenal loop samples collected (n = 5) were fixed (with 4% (*v*/*v*) buffered formaldehyde) on a slide (four sections for each sample), deparaffinized in xylene, rehydrated in ethanol, and stained with a combination of Alcian blue/periodic acid-Schiff (PAS). Slides were examined under a light microscope (BX3M series, Olympus Waltham, MA, USA). To count and measure the CellSens Standard Software was used. A total of 40 villi per cross-section (4 sections per sample) were measured for villus surface area (1 length and an average of 3 widths). Villus surface area was calculated using the following equation:

$$Villus\;surface\;area=2\pi \;x\frac{vw}{2}x\;VL$$

where *vw* is the average of three measurements of villus width, and VL is the villus length. Ten villi per sample were counted for number of goblet cells (blue–acidic, purple–mixed, and pink–neutral) and diameter. Ten circular crypts per sample were counted for Paneth cell number and diameter.

### 2.9. Statistical Analysis 

The results are all (unless specified otherwise) expressed as means ± Standard Error Means (SEM) in tables and heatmaps. Heatmaps were created in Microsoft Excel (Microsoft Corporation, Redmond, WA, USA) based on conditional formatting using color scales based on result means. Experiment groups were all assigned randomly ensuring even weight distribution to all groups. To assess distribution normality, the Shapiro-Wilk test was used. Normally distributed experimental group results were analyzed by one-way ANOVA. Analysis of Variance (ANOVA) was followed by a posthoc Duncan test using SPSS software version 27 (version 26.0, IBM, Armonk, NY, USA) with a significance value (*p* < 0.05).

## 3. Results

### 3.1. Polyphenol and Carbohydrate Analysis

The analysis revealed that the Grape Pomace (GP) extract had the most total polyphenols and highest (acid/neutral) detergent fiber content; whereas, the GPR corresponded to the highest amount of monomeric anthocyanins and non-fiber carbohydrates (*p* < 0.05, Table 2).

### 3.2. Hatchability and Body Weight

The hatchability with Grape Puree (GPR) and Grape Pomace (GP) was lower than expected. Whereas the more dilute treatment group-Grape Juice (GJ) resulted in 100% embryo survival as indicated in Table 3. No significant differences were observed in the body weight of the hatchlings.

### 3.3. Blood Glucose and Pectoral Glycogen Analysis

The different treatment groups did not result in significantly (*p* < 0.05) different values when compared to the controls and each other as seen in Table 3.

### 3.4. Duodenal Gene Expression

**Figure 2:**
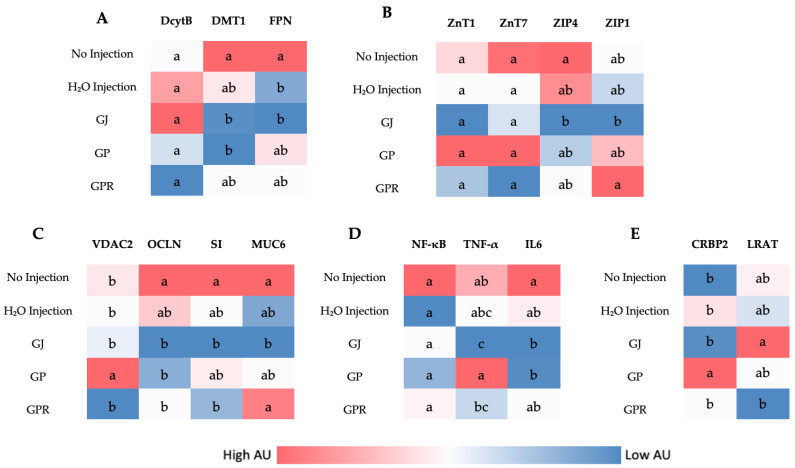
Heatmap showing the effect of intra-amniotic administration of grape juice (GJ), pomace (GP), and puree (GPR) on duodenal gene expression. (**A**)—iron metabolism-related genes, (**B**)—zinc metabolism-related genes, (**C**)—brush border membrane functionality genes, (**D**)—genes coding for inflammatory cytokines and (**E**)—Vitamin A metabolism-related genes. Values are in arbitrary units (AU). Genes not indicated by the same letter (a,b,c) are significantly different (*p* < 0.05). Key; Iron metabolism (DMT1: Divalent metal transporter 1; DcytB: Duodenal cytochrome b), Zinc metabolism (ZIP: Zrt-, Irt-like proteins; ZnT: zinc transporter), Vitamin A metabolism (CRBP2: Cellular retinol-binding protein; LRAT: Lecithin retinol acyltransferase), inflammatory cytokines (NF-κB: Nuclear factor-kappa beta; IL6: Interleukin 6; TNF-α: Tumor Necrosis Factor Alpha), Brush border membrane functionality (VDAC: Voltage-dependent anion channel; SI: Sucrose isomaltase; OCLN: Occludin; MUC6: Mucin).

Figure 2 illustrates the differences in gene expression of proteins related to Zn, Fe, Vitamin A metabolism, inflammatory cytokines, and BBM functionality. The duodenum is the main site of Zn, Fe, and Vitamin A absorption in *Gallus gallus* [50,51]. The grape fractions did not result in any significant differences in Zn metabolism-related genes (ZnT1, ZnT7, ZIP4, and ZIP1) between the different groups. Similarly, the expression of Fe metabolism-related genes (DcytB, DMT1, and Ferroportin), inflammation-related genes (*IL-6*, NF-κB, and TNF-α), and BBM functionality biomarkers (*OCLN*, *SI*, and *MUC6*) did not significantly change when comparing the H₂O Injection to the other treatment groups; however, the expression of *VDAC2* was upregulated in GP (6% grape pomace) compared to all other groups. Vitamin A metabolism-related gene *CRBP2* was upregulated in GP compared to all other groups, and *LRAT* was significantly different between GJ and GPR.

### 3.5. Liver Gene Expression

**Figure 3:**
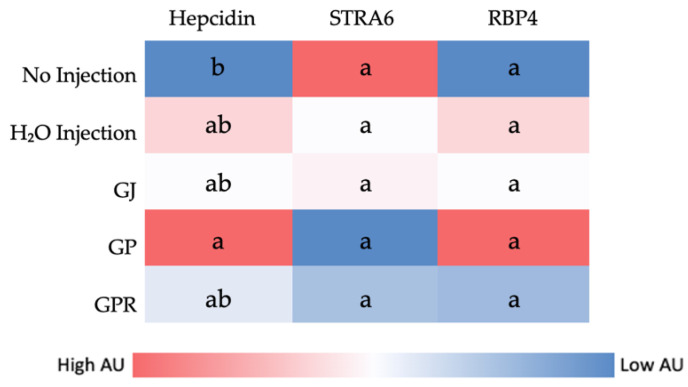
Heatmap showing the effect of intra-amniotic administration of grape juice (GJ), pomace (GP), and puree (GPR) on liver gene expression. Values are in arbitrary units (AU). Genes not indicated by the same letter (a,b) are significantly different (*p* < 0.05). Key; STRA6: Signaling receptor and transporter of retinol; RBP: Retinol-binding protein.

Figure 3 depicts no significant differences in gene expression of Vitamin A metabolism-related genes *RBP4*, *STRA6*, and iron metabolism-related protein Hepcidin (when compared to H₂O Injection) assessed in the liver.

### 3.6. Duodenal Morphometric Parameters

The villi surface area was significantly reduced with the intra-amniotic administration of GP when compared to the controls (No injection and H_2_O). Whereas no significant changes were observed in Paneth cell number or diameter between the treatment groups and the no injection control. A shorter crypt depth indicates increased villi change over rate. GP treatment corresponded to the smallest crypt depth whereas GPR was the largest (*p* < 0.05, Table 4).

The grape juice treated group showed the highest villi goblet cell number, significantly (*p* < 0.05) higher than the controls. Whereas the GP group was not significantly different from the (no injection) control but lower than GJ and GPR. Similarly, the goblet cell diameter was lower in GP when compared to GJ and GPR (Table 5).

Similar to the results of villi goblet cells, crypt goblet cells too were found to be lower (*p* < 0.05) in diameter in the GP when compared to GJ and GPR. Crypt goblet cell number was found to be the highest in the GJ group (Table 6).

### 3.7. Analysis of the Gut Bacterial Populations

**Figure 4:**
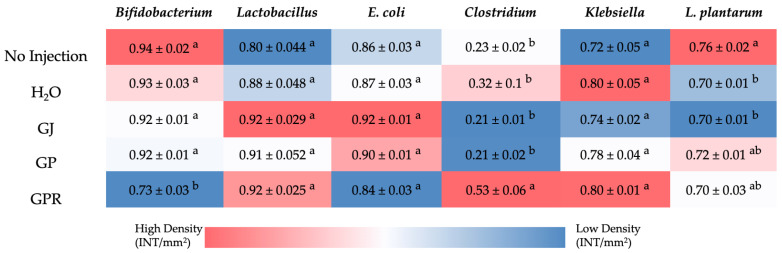
Heatmap showing the difference in cecal bacterial population abundances following intra-amniotic administration of grape juice (GJ), pomace (GP), and puree (GPR). Values are the means ± standard error means (n = 5); Values followed by a different letter indicate statistically significant differences assessed by ANOVA followed by Duncan posthoc test.

The 16s rDNA analysis of cecal bacterial populations showed no significant changes in the relative abundance of the *Lactobacillus*, *Klebsiella., E. coli,* and *L. plantarum* when compared to the H_2_O injection. However, GPR resulted in higher (*p* < 0.05) *Clostridium* numbers and lower *Bifidobacterium* numbers when compared to all other groups (Figure 4).

## 4. Discussion

In the present study, we have shown that the intra-amniotic administration of grape pomace, puree, and juice can alter duodenal morphology, gene expression, and specific cecal bacterial populations in-vivo (*Gallus gallus*). These alterations are in-line with previous literature, suggesting that grape pomace and puree may be included in food products and feed provided their concentration is limited. Grape juice corresponds to the lowest fiber and polyphenol content of the three treatment groups and was found to produce the most desirable results in comparison.

A recent meta-analysis of randomized control trials showed that whole grapes and grape products increased fasting blood glucose levels [52], whereas another comprehensive review on grape wines concluded no effect on blood glucose [53]. In this study, we found no significant changes in blood glucose nor stored glucose (in the form of glycogen) in pectoral muscles. In the case of the second review, wine processing reduced the available sugars to alcohol, thereby preventing the rise in blood sugar. Additionally, in this study, the sugars were probably too diluted to cause any significant spikes in blood glucose (Table 3). In addition, no differences were observed in the body weight of the hatchlings. Other animal studies on pigs [54] and broilers [55,56] have similarly reported no significant changes in body weight following daily dietary supplementation of either grape seed, grape marc meal extract, or grape pomace for four weeks or more.

The morphology of a healthy intestine corresponds to large villi, the presence of many mucin-producing goblet cells, and numerous anti-microbial peptide-producing Paneth cells. These parameters often indicate high nutrient absorption efficiency and strong pathogen defenses [57,58]. In the present study, the morphometric assessment of the duodenum revealed a reduction (*p* < 0.05) in the villus absorptive surface area in the group administered with grape pomace (GP) when compared to the controls (Table 4). A general trend of reduced villi surface area is observed in the grape-treated groups. At the same time, no significant changes were observed in villi goblet cell number/diameter nor Paneth cell number/diameter when compared to both the controls (Table 4 and Table 5). A similar reduction in duodenal villus height was observed in broilers following grape pomace dietary inclusion [55]. This suggests that the polyphenol “cocktail” found in grapes inhibits the proliferation of enterocytes [59,60]. Similarly, the *C. sativus* (saffron) floral bio-residue polyphenolic blend caused a dose-dependent decrease in villi surface area following intra-amniotic administration [31]. This may be due to the reduced digestibility of proteins. It has been shown that the reactive hydroxyl group of polyphenols interacts with the carbonyl group of proteins and forms undigestible complexes [55,61,62]. Proteins are essential for cell division and proliferation [63]. The interaction between polyphenols and proteins could explain the reduction in duodenal enterocyte proliferation and thereby, villus surface area. In this study, the total polyphenol concentration (Table 2) is inversely proportional to the surface area of villi (Table 4) and directly proportional to hatchability/survivability (Table 3).

This is the first study that investigated the effect of grapes on hepatic and brush border membrane micronutrient metabolism-related gene expression. For this investigation, we utilized a novel intra-amniotic approach using the embryonic phase of *Gallus gallus*. Previous studies have found the expression of pro-inflammatory, polyphenol absorption-related genes to be reduced and intestinal barrier integrity-related genes to be upregulated in mice and pigs when supplemented with grape polyphenols [54,64]. In the present study, we did not see any significant differences in iron (DMT1, FPN, and DcytB) and zinc (ZnT1, ZnT7, ZIP4, and ZIP1) metabolism-related genes in the treatment groups when compared to the controls. However, the expression of *CRBP2* (cellular retinol-binding protein 2) was significantly upregulated in the group treated with GP. *CRBP2* plays a crucial role in the intracellular transport of retinol and hence in retinoid signaling. It has been shown that resveratrol (a bioactive stilbene found in grapes and grape pomace) can upregulate *CRBP2* expression in the thyroid cancer cell line (THJ-11T) [65]. It is hypothesized that resveratrol (in GP) led to the increased expression of CRBP2. Similarly, the expression of *VDAC2* (Voltage-dependent anion-selective channel 2) was upregulated in the GP-administered group when compared to the others. *VDAC2* is abundant in the outer membrane of the mitochondria and has an anti-apoptotic role [66]. Previously, resveratrol has been shown to protect the mitochondria against age-related dysfunction [67]. Activation of *VDAC2* is perhaps one such mechanism. Surprisingly, no significant changes were observed in the expression of inflammatory cytokines as is expected with grape polyphenols treatment. A longer duration of consumption of grape polyphenols may be required to see the anti-inflammatory effects. Additionally, no significant differences were observed in genes assessed in the liver (Hepcidin coding gene, *STRA6*, and *RBP4*) as seen in Figure 3.

The relative abundance of bacterial populations assessed in the cecum remained largely unchanged with the intra-amniotic administration of grape puree, pomace, and juice compared to the controls. Similar results were reported in broilers fed 15 g/kg grape pomace dietary inclusion for 42 days. The study reported no significant differences (*p* < 0.05) in the gene copy number of *Lactobacillus* spp., *Enterococcus* spp., *E. coli*, *Campylobacter jejuni*, *S. aureus* nor *C. perfringens* in cecum between the groups that were fed grape pomace and the control [56]. These results suggest that the dietary inclusion of grape pomace and grape juice maintains the gut microbiota without disturbing it. However, the GPR treatment resulted in a lower relative abundance of beneficial bacteria *Bifidobacterium* and a higher abundance of *Clostridium.* This finding could be explained by the presence of a high concentration of non-fibrous carbohydrates (NFC, Table 2) in the GPR group relative to the others. NFCs include sugars, starches, organic acids, and pectin. Dietary sugars are shown to fuel the proliferation of members of the genus *Clostridium* [68,69,70]. These changes in cecal bacterial populations, however, appear not to correlate with changes in duodenal morphology and gene expression. This suggests that overall, grape puree did not cause any severe detrimental effects to the gut based on the parameters assessed in this study.

## 5. Conclusions

This is the first study to comparatively assess grape pomace, juice, and puree on various aspects of intestinal health in-vivo. The inclusion of grape pomace (a low-cost by-product of the wine and grape juice industry) in feed and food could not only offset the negative environmental impact but also reduce their manufacturing cost without negatively affecting consumer intestinal health. The grape juice fraction seems to have the most desirable effect on gut health; however, a lower concentration of grape pomace and puree may generate similar effects. This is an exploratory interventional study; further long-term studies are now warranted to confirm the findings reported here.

## Figures and Tables

**Table 1:** The sequences of the primers (both forward and reserve) used in this study are displayed. GenInfo Identifier number and base-pair lengths have also been specified. All were assessed in the duodenum except for those represented by * these were assessed in the liver.

Analyte	Forward Primer (5′-3′)	Reverse Primer (5′-3′)	Base Pairs Length	GI Number
Iron Metabolism				
DMT1	TTGATTCAGAGCCTCCCATTAG	GCGAGGAGTAGGCTTGTATTT	101	751817
Ferroportin	CTCAGCAATCACTGGCATCA	ACTGGGCAACTCCAGAAATAAG	98	423984
DcytB	CATGTGCATTCTCTTCCAAAGTC	CTCCTTGGTGACCGCATTAT	103	20380692
Hepcidin *	AGACGACAATGCAGACTAACC	CTGCAGCAATCCCACATTTC	132	SAMN08056490
Immune Response				
NF-κB	CACAGCTGGAGGGAAGTAAAT	TTGAGTAAGGAAGTGAGGTTGAG	100	396033
*IL-6*	ACCTCATCCTCCGAGACTTTA	GCACTGAAACTCCTGGTCTT	105	395337
TNF-𝛂	GACAGCCTATGCCAACAAGTA	TTACAGGAAGGGCAACTCATC	109	374125
Zinc Metabolism				
ZnT1	GGTAACAGAGCTGCCTTAACT	GGTAACAGAGCTGCCTTAACT	105	423089
ZnT7	GGAAGATGTCAGGATGGTTCA	CGAAGGACAAATTGAGGCAAAG	87	424464
ZIP4	TCTCCTTAGCAGACAATTGAG	GTGACAAACAAGTAGGCGAAAC	95	107050877
ZIP1	TGCCTCAGTTTCCCTCAC	GGCTCTTAAGGGCACTTCT	144	121112053
Vitamin A Metabolism				
*CRBP2*	GGCTACATGGTTGCACTAGACA	AACCACCCGGTTATCGAGTC	195	NM_001277417.1
*LRAT*	GATTTTGCCTATGGCGGCAG	TTGTCGGTCTGGAAGCTGAC	197	22403
STRA6 *	GTGCGCTGAACTTTGTCTGC	TTCTTCCTGCTCCCGACCT	116	415301
RBP4 *	TGCCACCAACACAGAACTCTC	CTTTGAAGCTGCTCACACGG	149	396454
BBM Functionality				
*VDAC2*	CAGCACTCGCTTTGGAATTG	GTGTAACCCACTCCAACTAGAC	99	395498
*SI*	CCAGCAATGCCAGCATATTG	CGGTTTCTCCTTACCACTTCTT	95	425007
*OCLN*	GTCTGTGGGTTCCTCATCGT	GTTCTTCACCCACTCCTCCA	124	396026
*MUC6*	CCAACTTGCAGTGTTCCAAAG	CTGACAGTGTAGAGCAAGTACAG	106	414878
18s rRNA	GCAAGACGAACTAAAGCGAAAG	TCGGAACTACGACGGTATCT	100	7262899

DMT1: Divalent metal transporter 1; Dcytb: Duodenal cytochrome b; NF-κB: Nuclear factor-kappa beta; IL6: Interleukin 6; TNF-α: Tumor Necrosis Factor Alpha; ZIP: Zrt-, Irt-like proteins; ZnT: zinc transporter; CRBP2: Cellular retinol-binding protein; LRAT: Lecithin retinol acyltransferase; STRA6: Signaling receptor and transporter of retinol; RBP: Retinol-binding protein; VDAC: Voltage-dependent anion channel; SI: Sucrose isomaltase; OCLN: Occludin; MUC6: Mucin.

**Table 2:** Total polyphenol content (TPC), monomeric anthocyanins (MA), acid detergent fiber (ADF), neutral detergent fiber (NDF), and non-fiber carbohydrates (NFC) were estimated in the different grape fractions as appropriate.

Sample	TPC (mg/g GAE)	MA (CE/g)	ADF (%/DM)	NDF (%/DM)	NFC (%/DM)
GJ	2.4 ± 0.00 ^c^	858 ± 256 ^c^	NA	NA	NA
GP	11.6 ± 0.05 ^a^	2353 ± 159 ^b^	41.1	43.7	29.4
GPR	7.1 ± 0.30 ^b^	2544 ± 91 ^a^	5.7	6.3	82.8

Values are the means ± standard deviation, superscripts in the same column indicate a significant difference (*p* < 0.05). ADF–cellulose, lignin, and insoluble minerals; NDF-cellulose, lignin, insoluble minerals, and hemicellulose; NFC-sugars, starches, organic acids, and pectin. GAE–gallic acid equivalence; CE-cyanidin-3-glucoside equivalents; DM–dry matter.

**Table 3:** Hatchability, body weight, blood glucose, and pectoral glycogen values.

Treatment Group	Hatch/Injected	Body Weight (g)	Blood Glucose (mg/dL)	Glycogen (mg/g)
No Injection	9/10	40.8 ± 1.2 ^a^	254 ± 24 ^a^	0.40 ± 0.10 ^a^
H_2_O	9/10	38.3 ± 4.3 ^a^	234 ± 11 ^a^	0.30 ± 0.09 ^a^
GJ	13/13	38.9 ± 1.7 ^a^	226 ± 12 ^a^	0.30 ± 0.06 ^a^
GP	6/10	36.8 ± 1.2 ^a^	314 ± 0.1 ^a^	0.23 ± 0.11 ^a^
GPR	9/12	39.6 ± 0.8 ^a^	226 ± 12 ^a^	0.32 ± 0.10 ^a^

Values are the means ± SEM (n = 8). Treatment groups are all indicated by the same letter ^a^ hence statistically insignificant (*p* < 0.05).

**Table 4:** Effect of the intra-amniotic administration of grape juice (GJ), pomace (GP), and puree (GPR) on the duodenal villi surface area, crypt depth, Paneth cell number, and diameter.

Treatment Group	Villi Surface Area (µm^2^)	Crypt Depth (µm)	Paneth Cell Number	Paneth Cell Diameter (µm)
No Injection	164.6 ± 7.7 ᵃ	22.1 ± 0.8 ^a^	1.2 ± 0.03 ᵃ	1.4 ± 0.02 ᵇ
H_2_O	161.0 ± 3.8 ᵃᵇ	21.9 ± 0.7 ^a^	1.0 ± 0.01 ᵇ	1.5 ± 0.02 ᵃ
GJ	153.3 ± 3.9 ᵃᵇᶜ	16.2 ± 0.6 ᵇ	1.3 ± 0.03 ᵃ	1.4 ± 0.02 ᵇ
GP	145.4 ± 4.3 ᶜ	13.8 ± 0.5 ᶜ	1.3 ± 0.04 ᵃ	1.4 ± 0.02 ᵇ
GPR	148.8± 3.9 ᵇᶜ	15.4 ± 0.5 ^bc^	1.3 ± 0.04 ᵃ	1.4 ± 0.02 ᵇ

Values are the means ± SEM (n = 5). ^a–c^ Treatment groups not indicated by the same letter are significantly different (*p* < 0.05).

**Table 5:** Effect of the intra-amniotic administration of grape juice (GJ), pomace (GP), and puree (GPR) on the duodenal villus goblet cell type, number, and diameter.

Treatment Group	Villi Goblet Cell Diameter (µM)	Total Villi Goblet Cell Number	Villus Goblet Cell Type-Number		
			Acidic	Neutral	Mixed
No Injection	3.5 ± 0.07 ᵃ	20.1 ± 0.60 ᵈ	18.3 ± 0.58 ᵈ	0.06 ± 0.02 ᵇ	1.8 ± 0.14 ᶜ
H_2_O	3.2 ± 0.06 ᵇᶜ	33.2 ± 0.73 ᶜ	29.9 ± 0.69 ᶜ	0.22 ± 0.04 ᵇ	3.2 ± 0.20 ᵃ
GJ	3.3 ± 0.07 ᵃᵇ	41.7 ± 0.95 ᵃ	39.0 ± 0.87 ᵃ	0.14 ± 0.04 ᵇ	2.6 ± 0.23 ᵇ
GP	3.0 ± 0.06 ᶜ	21.6 ± 1.22 ᵈ	18.9 ± 1.20 ᵈ	0.62 ± 0.09 ᵃ	2.1 ± 0.14 ᵇᶜ
GPR	3.3 ± 0.07 ᵇ	38.4 ± 0.98 ᵇ	34.7 ± 0.87 ᵇ	0.21 ± 0.05 ᵇ	3.5 ± 0.26 ᵃ

Values are the means ± SEM (n = 5). ^a–d^ Treatment groups not indicated by the same letter are significantly different (*p* < 0.05).

**Table 6:** Effect of the intra-amniotic administration of grape juice (GJ), pomace (GP), and puree (GPR) on the duodenal crypt goblet cell type, number, and diameter.

Treatment Group	Crypt Goblet Cell Diameter (µM)	Total Crypt Goblet Cell Number	Crypt Goblet Cell Type-Number		
			Acidic	Neutral	Mixed
No Injection	3.0 ± 0.05 ᵇ	7.0 ± 0.24 ᶜ	5.8 ± 0.20 ^c^	0.02 ± 0.02 ᶜ	1.2 ± 0.1 ᶜ
H_2_O	2.9 ± 0.05 ᵇ	8.6 ± 0.32 ᵇ	6.9 ± 0.28 ^b^	0.13 ± 0.03 ^b^	1.5 ± 0.1 ^bc^
GJ	2.9 ± 0.07 ᵇ	10.6 ± 0.36 ᵃ	8.0 ± 0.29 ^a^	0.39 ± 0.05 ^a^	2.2 ± 0.1 ^a^
GP	2.7 ± 0.05 ᶜ	8.1 ± 0.27 ᵇ	6.4 ± 0.25 ^bc^	0.0 ± 0.0 ^c^	1.7 ± 0.1 ^b^
GPR	3.3 ± 0.05 ᵃ	8.3 ± 0.28 ᵇ	6.7 ± 0.21 ᵇ	0.0 ± 0.0 ^c^	1.6 ± 0.1 ᵇ

Values are the means ± SEM (n = 5). ^a–c^ Treatment groups not indicated by the same letter are significantly different (*p* < 0.05).

## Data Availability

Not applicable.
